# Does the *CAPON* Gene Confer Susceptibility to Schizophrenia?

**DOI:** 10.1371/journal.pmed.0020348

**Published:** 2005-10-25

**Authors:** Sharon L Eastwood

## Abstract

Eastwood discusses a new study in *PLoS Medicine* that suggests that overexpression of the *CAPON* gene, leading to disruption of NMDA receptor function, may be important in the etiology of severe mental illnesses.

A major breakthrough towards a greater understanding of the pathophysiology of severe mental illness came with the recent identification of potential susceptibility genes for schizophrenia and bipolar disorder, the two main diagnostic categories of functional psychoses. Although it has been known for many years that schizophrenia and bipolar disorder tend to run in families, and twin and adoption studies have clearly established the heritability of both disorders, no single “gene for schizophrenia” or “gene for bipolar disorder” appears to exist. Instead, the pattern of inheritance suggests that these common mental illnesses are caused by many different genes of small effect, acting together to confer susceptibility.

For schizophrenia, several candidate susceptibility genes have been identified, but for all except one of them, the single-nucleotide polymorphisms found to be associated with the disorder are noncoding. Hence, changes in the expression of the genes (for example, in terms of their splicing or relative abundance), rather than an amino acid alteration, are thought to underlie their genetic association [1]. To investigate this possibility further, studies of gene expression in postmortem brains are required.

## A New Gene Expression Study

In this month's *PLoS Medicine*, Linda Brzustowicz and colleagues present the results of their study of one potential schizophrenia susceptibility gene, *CAPON* (carboxyl-terminal PDZ ligand of neuronal nitric oxide synthase), using postmortem tissue samples of the dorsolateral prefrontal cortex, a pathological “hotspot” identified in studies of schizophrenia [2].

**Figure 1:**
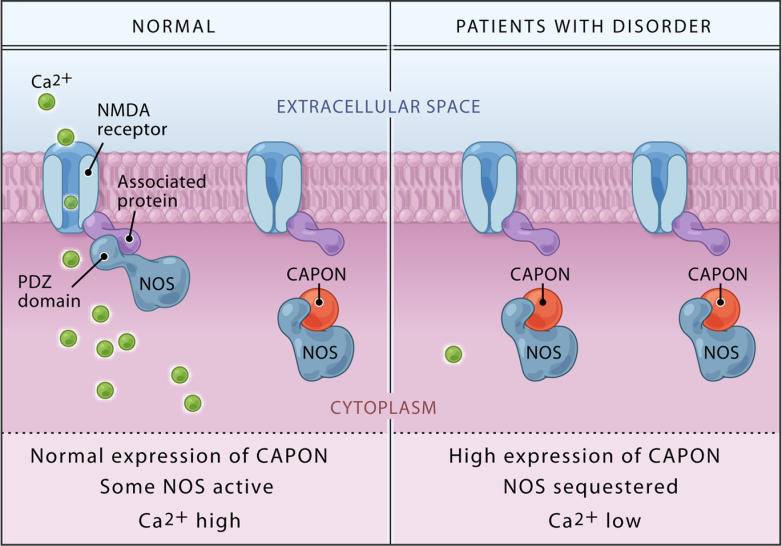
CAPON Binds Nitric Oxide Synthase, Regulating NMDA Receptor–Mediated Glutamate Neurotransmission Binding of CAPON results in a reduction of NMDA receptor/nitric oxide synthase (NOS) complexes, leading to decreased NMDA receptor–gated calcium influx and a catalytically inactive nitric oxide synthase. Overexpression of either the full-length or the novel shortened CAPON isoform as reported by Brzustowicz and colleagues is, therefore, predicted to lead to impaired NMDA receptor–mediated glutamate neurotransmission. (Illustration: Bang Wong, http://www.clearscience.info)

The gene for *CAPON* is located on Chromosome 1q22, a locus of interest initially identified from linkage studies of schizophrenia. Two studies, including one by Brzustowicz and colleagues, subsequently detected an association between single-nucleotide polymorphisms within the *CAPON* gene and the occurrence of schizophrenia [3,4]. The role of CAPON in binding nitric oxide synthase (Figure 1) places it at the centre of regulation of N-methyl-D-aspartate (NMDA) receptor–mediated glutamate neurotransmission, abnormalities of which have long been proposed to be involved in schizophrenia, lending credence to *CAPON* as a susceptibility gene for the disorder. However, as sequencing of *CAPON* failed to reveal a coding mutation, Brzustowicz and colleagues went on to examine whether the expression of the gene is altered in schizophrenia.

First, using standard techniques, the authors identified a novel *CAPON* mRNA transcript, and verified that the predicted shortened protein was expressed in the brain. In order to continue on with this work, and determine if *CAPON* expression was altered in schizophrenia, they used RNA samples isolated from the dorsolateral prefrontal cortex of psychiatrically normal individuals, individuals with schizophrenia, and individuals with bipolar disorder. These samples were provided by the Stanley Medical Research Institute (http://www.stanleyresearch.org/programs/brain_collection.asp). Although the authors' primary interest was schizophrenia, as a condition of use, the Stanley Medical Research Institute required investigators to be blinded to diagnosis, and, hence, the experiments were run on individuals with bipolar disorder as well.

Using quantitative real-time polymerase chain reaction, the authors found that while the mRNA for the full-length CAPON protein was unchanged in either disorder, for both schizophrenia and bipolar disorder, mRNA expression of the novel shortened CAPON isoform was increased compared to the psychiatrically normal controls. Antipsychotic drug treatment received by the patients did not appear to underlie the authors' findings, and genotyping revealed that individuals with one or two copies of alleles previously identified as associated with schizophrenia had higher levels of the novel CAPON isoform mRNA. The authors concluded that overexpression of either CAPON isoform would be expected to disrupt NMDA receptor function, and that the results of their study not only added support to the role of *CAPON* in schizophrenia, but also implicated the gene in the aetiology of bipolar disorder.

## Limitations of the Study

All postmortem studies such as this are limited by the suitability of the material available, and the brain series from which they came. The Stanley Medical Research Institute series used by the authors is well matched for variables known to influence studies of gene expression, and the results of the study are likely to be genuine. However, replication in another brain series should be attempted. In addition, as this was a homogenate-based study of one brain region, future studies should examine other areas, and also use additional techniques (such as in situ hybridization histochemistry) to determine whether expression is altered in all or only specific neuronal populations. As the authors were limited to examining RNA, it will also be interesting to determine if expression of the encoded shortened CAPON protein is similarly increased in schizophrenia and bipolar disorder. Lastly, functional in vitro studies examining the knock-on effects of overexpression of the shortened CAPON protein may be especially useful for understanding the function of this novel isoform and the pathophysiological consequences of the authors' findings.

## Conclusion

This study is an example of one approach that may be used to help us begin to understand how genes, such as *CAPON*, may confer risk for disease susceptibility. The paper also highlights the growing awareness that although, classically, schizophrenia and bipolar disorder have been conceptualised as separate disorders, it may be more appropriate to envisage them as phenotypical extremes of a continuum of disease caused by an overlapping set of genes [5]. Although the findings of this study are not of immediate clinical relevance, a better understanding of the genes and pathways that are involved will ultimately lead to the development of more effective treatments for both disorders.

## Abbreviations

CAPON: carboxyl-terminal PDZ ligand of neuronal nitric oxide synthase
NMDA: N-methyl-D-aspartate
